# Microanalysis Characterization and Immunomodulatory Effect for Selenium-Enriched Polysaccharide from *Morchella esculenta* (L.) Pers.

**DOI:** 10.3390/molecules28072885

**Published:** 2023-03-23

**Authors:** Lijuan Qian, Mengxiang Du, Xiaoyan Yang, Qian Wang, Shengwei Huang, Yuhan Ma, Yujun Sun

**Affiliations:** 1 College of Agriculture, Anhui Science and Technology University, Fengyang 233100, China; 2 College of Life and Health Sciences, Anhui Science and Technology University, Fengyang 233100, China

**Keywords:** *Morchella esculenta* (L.) Pers., selenium-enriched polysaccharide, physicochemical composition, structural characterization, immunomodulation, toll-like receptor 4

## Abstract

*Morchella esculenta* (L.) Pers., referred to as *Morel*, is a medicinal and edible homologous fungus, which contains many bioactive substances. In *Morel*, polysaccharides are the most abundant and have various bioactivities. In the present work, two novel polysaccharides, Se-MPS and MPS, were prepared and purified from selenium-enriched (Se-enriched) and common *Morel* mycelia, respectively, and their structural and immunomodulatory properties were evaluated. The results show that Se-enriched treatment significantly changed the polysaccharides’ chemical composition, molecular weight, and sugar chain configuration. In addition, the Se-enriched treatment also improved the polysaccharides’ fragmentation and thermal stability. Importantly, Se-enriched *Morel* polysaccharide (Se-MPS) could significantly enhance phagocytosis of RAW 264.7 macrophage cells and, remarkably, activate their immune response via activating the TLR4-TRAF6-MAPKs-NF-κB cascade signaling pathway, finally exerting an immunomodulatory function. Based on these findings, selenium-enriched *Morel* polysaccharide appears to have more potential for development and utilization in functional foods or medicines than ordinary *Morel* polysaccharide.

## 1. Introduction

*Morel* is a precious edible or medicinal mushroom highly valued worldwide for its unique sensory properties and high nutritional value [1]. Since *Morel* is rich in unique aroma, delicious taste, delicate flavor, and meaty texture, it is used in cooking delicacies [2]. Additionally, *Morels* have been used as traditional medicine for centuries due to their ability to prevent or treat many diseases [3]. Microbial resources including Morels serve as natural resources and gold mines for therapeutics, supplements and nutraceuticals discovery [4]. It is reported that the pharmacological activity of *Morel* is related to its various chemical components, including polysaccharides, amino acids or proteins, lipids, vitamins, sterols, and organic acids [1]. These natural products are widely used as botanical medicines and nutraceuticals to help prevent a variety of diseases, including cancer, cardiovascular disease and neurodegenerative health conditions [4]. *Morel* polysaccharides (MPS), in particular, have been proven to exhibit anti-tumor, antibacterial, and immune-enhancing effects and have received more and more attention from researchers [5]. As a result, *Morel* polysaccharide has considerable research and economic value, regardless of their application in healthcare or medicine.

Selenium (Se) is an essential trace mineral for the body’s proper functioning [6]. It plays a crucial role in maintaining a healthy immune system and protecting cells from oxidative stress [6]. There is also some evidence to suggest that Se may have a protective effect against certain types of cancer [7]. Usually, selenium exists as selenium acid, selenite, or elemental selenium in nature. However, the inorganic form of selenium can be converted into an organic form in various organisms by binding to polysaccharides, polypeptides, or proteins [8,9]. The importance of Se and MPS to human health cannot be overstated, and therefore, Se-MPS as a nutritional supplement can not only optimize the physiological and pharmacological functions of two components, but also reduce the intrinsic limitations of selenium and polysaccharides in processing applications such as their low solubility and bioavailability [10]. The physicochemical properties, bioactivities, and structural characterization of MPS have been thoroughly studied. However, few studies have been conducted on Se-MPS, which dramatically limits its development and utilization.

Immunomodulation is one of the typical bioactivities of edible fungal polysaccharides, and many edible fungal polysaccharides have been reported to exert immunomodulatory activity. For example, a polysaccharide isolated from the fruiting body of *Inonotus obliquus* was reported to induce NO/ROS production, TNF-α secretion, and phagocytic uptake in macrophages [11]. Additionally, it could modulate immune responses by promoting the activation of macrophages via MAPK and NF-κB pathways. Similarly, a water-soluble glucan polysaccharide from *Flammulina velutipes* mycelium could promote NO production, IL-1 production, and TNF-α secretion in macrophages [12]. Meanwhile, a study by Su et al. revealed that *Morchella conica* polysaccharide could regulate NO production in macrophages and modulate innate immunity at specific concentrations [13]. However, Se-enriched edible fungal polysaccharides, particularly Se-MPS, have not been studied for their immunomodulatory activity. It is necessary to conduct more investigation to improve understanding of the immunological activity of Se-enriched edible fungal polysaccharides. Aiming at this issue, we prepared two polysaccharides (Se-MPS and MPS) and investigated their structural and immunomodulatory properties. In addition, the primary mechanism of immune activation was further investigated.

## 2. Results and Discussion

### 2.1. Preliminary Composition Analysis

A significant increase was observed in yields for Se-enriched (8.74 ± 0.14%) and non-Se-enriched (15.74 ± 0.11%) crude polysaccharides compared to previously published data from *L. edodes* mycelium polysaccharides [14]. According to reports, organoselenium contributed to higher selenopolysaccharide yields [15], suggesting that Se in Se-MPS may mainly exist as organic selenium. From the yield comparison, the selenium enrichment could also affect crude *Morel* polysaccharide yield. After purification by using Sepharose FF gels, only 0.5 M NaCl eluted fractions delivered high yields (11.68 ± 0.25% and 14.65 ± 0.18%, respectively), while the yields of other eluted fractions were less than 1% (Figure S1). Therefore, we only performed further analysis on the 0.5 M NaCl eluted fraction. Preliminary composition analysis (Table 1) showed that the prepared MPS and Se-MPS contained high total sugar content and trace amounts of uronic acid and protein, indicating that the two purified fractions are acidic polysaccharides carrying a small amount of binding protein [16]. Further, Se-MPS had lower uronic acid levels than MPS, indicating that selenization degrades uronic acid [8]. Notably, the selenium content of Se-MPS after the cultivation of the selenium enrichment step was much higher than that of MPS, indicating the effectiveness of our artificial selenizing procedure [17].

**Figure 1:**
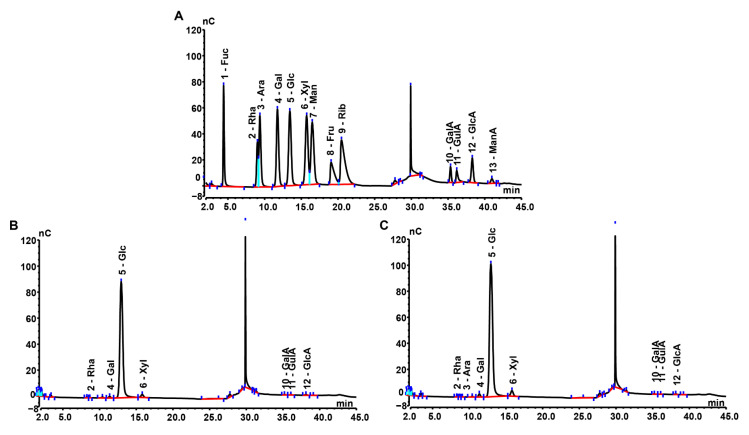
High-performance anion-exchange chromatography of monosaccharide compositions. (**A**) Monosaccharide standards, (**B**) MPS, and (**C**) Se-MPS.

**Figure 2:**
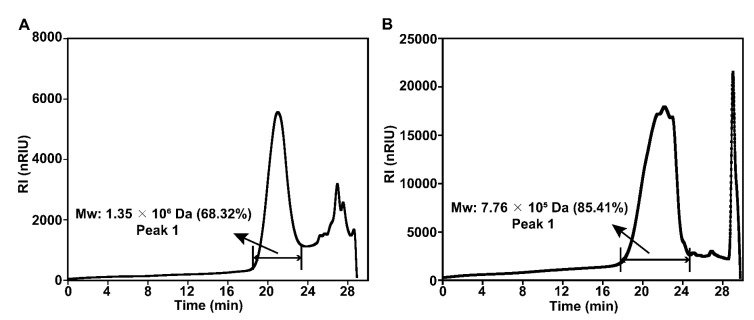
HPGPC profiles of MPS (**A**) and Se-MPS (**B**).

Monosaccharide composition analysis (Figure 1) showed that MPS was composed of Rha, Gal, Glc, Xyl, GalA, GluA, and GlcA, with a molar ratio of 0.35:1.70:171.00:1.29:4.54:2.06:4.30, whereas Se-MPS was composed of Rha, Ara, Gal, Glc, Xyl, GalA, GluA, and GlcA, with a molar ratio of 0.26:0.23:4.54:273.30:3.14:4.18:1.40:3.62. This result indicated that the selenium-enriched treatment changed the monosaccharide composition of MPS, which was consistent with the results reported by Zhu et al. [8]. As well as different molar ratios of monosaccharides, Ara was also present in Se-MPS, suggesting that artificial selenization might produce new monosaccharides in MPS [8]. Interestingly, Glc was the dominant monosaccharide (≥90%) in both polysaccharides, which was consistent with previous reports [18,19]. These results indicated that artificial selenization might not change the proportion of dominant monosaccharides in MPS. As shown in Figure 2, both polysaccharides were inhomogeneous, thus their structures could not be analyzed in detail [16]. The Mws of MPS and Se-MPS were 1.35 × 106 Da (68.32%) and 7.76 × 105 Da (85.41%), respectively, suggesting that selenium enrichment reduced the relative density of polysaccharides and results in a lower Mw [20]. As polar macromolecular polymers, polysaccharides have intermolecular or intramolecular hydrogen bond interactions between the many hydroxyl groups in the chains, which can influence their bioactivity. In contrast, polysaccharides with small Mws can usually exhibit stronger bioactivity because they can freely cross biomembranes to escape the pressure of the immune system. As a result, Se-MPS with a lower Mw might have a competitive advantage in certain bioactivities.

### 2.2. Chemical Characterization

**Figure 3:**
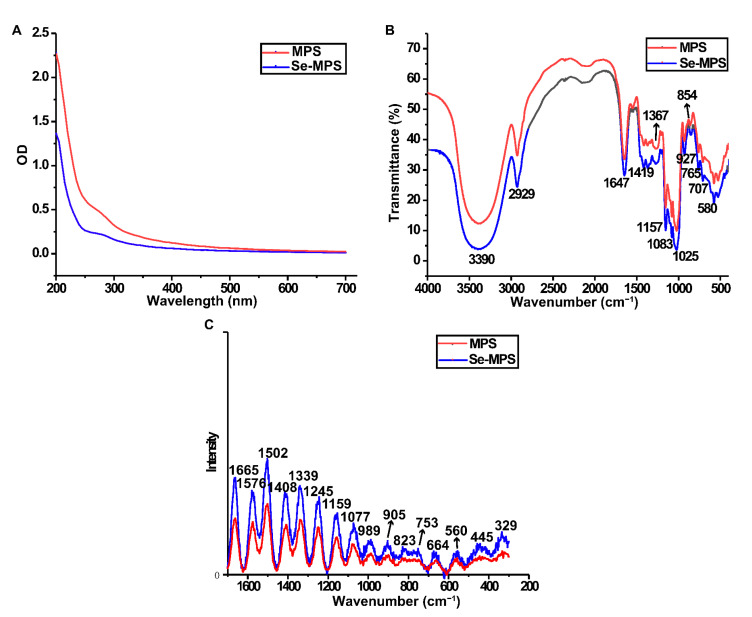
UV-vis (**A**), FT-IR (**B**), and Raman (**C**) spectra of MPS and Se-MPS.

The UV-vis spectrum (Figure 3A) indicated that both polysaccharides contained trace amounts of protein (weak absorption peaks at 280 nm), and these findings were also following preliminary composition analysis.

Se-MPS exhibited a similar FT-IR spectrum to MPS (Figure 3B). The strong absorption peaks at 3390 and 2929 cm^−1^ were attributed to O-H and C-H stretching vibrations, respectively [21]. The absorption peak at 1647 cm^−1^ and the dense absorption peak around 1419 cm^−1^ could be attributed to O-C=O antisymmetric and symmetric stretching vibrations, indicating that they may contain uronic acid [22]. A typical absorption peak at 1200–1000 cm^−1^ indicated the presence of a pyranose ring [23]. Low-intensity absorption peaks at 927 and 854 cm^−1^ indicated the presence of α-glycosidic linkages [14]. In agreement with Wang and Qiu et al. [24,25], these data also demonstrated that artificial selenium did not change the functional groups in MPS. Additionally, the absorption peak at 1025 cm^−1^ could be explained by the stretching vibration of O-Se-O bond [26], which preliminarily demonstrated the existence of organoselenium in Se-MPS.

Raman spectrum can indicate potential information of some functional groups and glycosidic bond types in MPS and Se-MPS. The peak at 1665 cm^−1^ (Figure 3C) indicated protein absorption [27]. Raman bands in the range of 1450–1000 cm^−1^ indicated the presence of polysaccharides, while Raman bands in the range 900–1200 cm^−1^ corresponded to the coupled C-O/C-C stretching and C-OH bending vibration [28]. A band at 800–830 cm^−1^ was associated with an anomeric structure surrounding the glycosidic bond of polysaccharides [29]. The absorption peaks around 989 and 560 cm^−1^ could be assigned to the presence of α-1,3-glucan, whereas the absorption peak around 445 cm^−1^ indicated the presence of β-1,3-glucan [28]. Interestingly, a stronger absorption peak at nearly 753 cm^−1^ in Se-MPS was presented, which reflected the characteristic absorption peak of Se=O bond and further confirmed the presence of organoselenium-modification [8].

**Figure 4:**
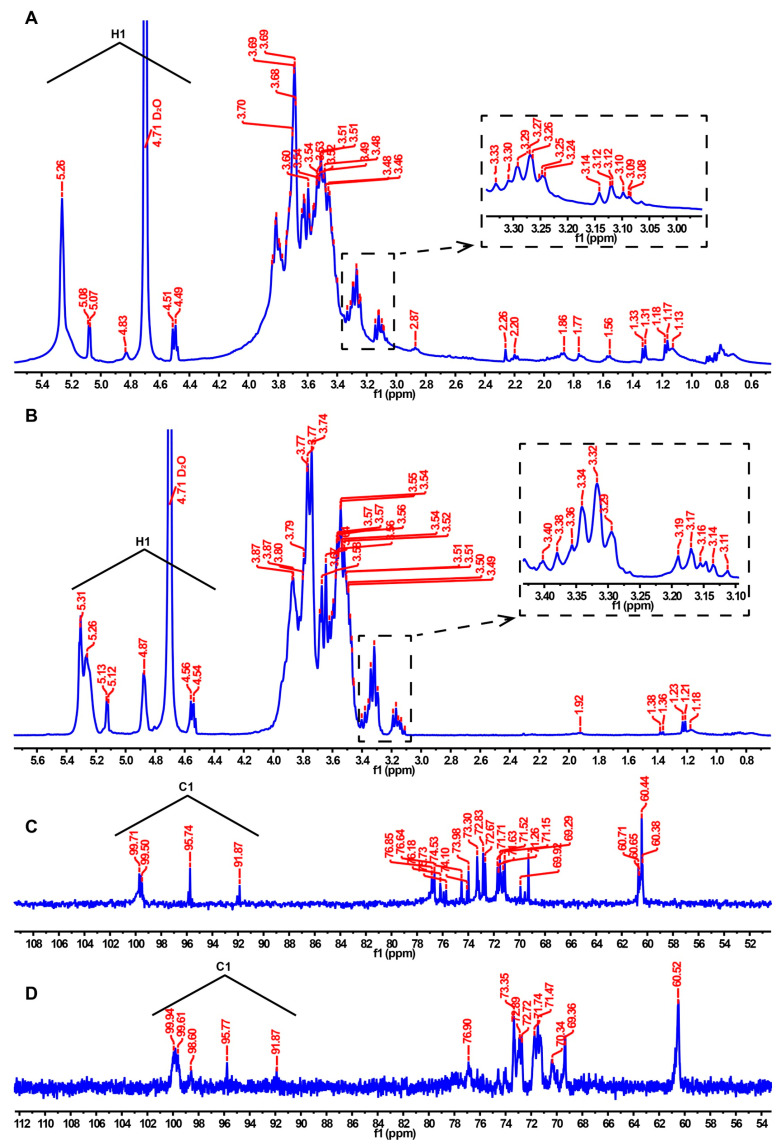
^1^H NMR and ^13^C NMR spectra of MPS (**A**,**C**) and Se-MPS (**B**,**D**).

NMR analysis was used to compare the differences in the structural properties of Se-MPS and MPS. The ^1^H NMR spectrum is mainly used to reflect the configuration information of glycosidic bonds. Generally, those with a chemical shift value greater than 4.95 ppm are α-type glycosides, and those with a chemical shift value less than 4.95 ppm are β-type [8]. The chemical shift of the anomeric carbon occurs at 90–110 ppm in ^13^C NMR spectroscopy, which is used to determine the number of sugar residues [8]. The ^1^H NMR spectrum of MPS (Figure 4A) exhibited seven signal peaks in the range of 4.5–5.5 ppm, but the signal peaks at 5.26, 5.08, 4.83, 4.71 (D_2_O suppression), and 4.50 were the prominent signal peaks according to the integrated area. Similarly, the ^1^H NMR spectrum of Se-MPS (Figure 4B) showed eight signal peaks, and the signal peaks at 5.31, 5.26, 5.13, 4.87, 4.71 (D_2_O suppression), and 4.55 were prominent. The signal at 5.31 of Se-MPS was tentatively deduced to be the H-1 of 1,4-α-d-Glcp [30]. The signals at 5.26 of MPS and Se-MPS were tentatively deduced to be the H-1 of 4-α-GalpA [31]. The signal at 5.13 of Se-MPS was tentatively deduced to be the H-1 of 1,2-α-Rhap [31]. The signal at 5.08 of MPS was tentatively deduced to be the H-1 of 1, 4-α-GalpA [32]. The signal at 4.87 of Se-MPS was tentatively deduced to be the H-1 of 1-α-d-Galp [33]. The signal at 4.83 of MPS was tentatively deduced to be the H-1 of 1-β-d-Galp [33]. The signals at 4.50 and 4.55 of MPS and Se-MPS were tentatively deduced to be the H-1 of 1-β-d-Glcp [33]. ^1^H NMR spectrum showed that both polysaccharides had α- and β-glycosidic bond configurations. Furthermore, the selenization modification elevated the chemical shift of Se-MPS and generated new glycosidic bonds, as shown by comparing the chemical shift values of the ^1^H NMR spectra. Further ^13^C NMR spectra also confirmed this result, and a new signal peak appeared at 98.60 ppm for Se-MPS (Figure 4C,D). Generally, if the ^13^C-terminal signal appears in a region greater than 100 ppm, the monosaccharide residue type may be β-d or α-l. Otherwise, it may be α-d or β-l [8]. According to ^13^C NMR spectroscopy, MPS might have four monosaccharide residues, whereas Se-MPS might have five monosaccharide residues, all of them might be β-d or α-l type. The signal at 99.94 of Se-MPS was tentatively deduced to be the C-1 of 1,4-α-d-Glcp [30]. The signals at 91.87 of MPS and Se-MPS were tentatively deduced to be the C-1 of 4-α-GalpA [31]. The signal at 99.61 of Se-MPS was tentatively deduced to be the C-1 of 1,2-α-Rhap [31]. The signal at 99.71 of MPS was tentatively deduced to be the C-1 of 1, 4-α-GalpA [32]. The signal at 98.60 of Se-MPS was tentatively deduced to be the H-1 of 1-α-d-Galp [33]. The signal at 99.50 of MPS was tentatively deduced to be the H-1 of 1-β-d-Galp [33]. The signals at 95.74 and 95.77 of MPS and Se-MPS were tentatively deduced to be the C-1 of 1-β-d-Glcp [33]. It was noteworthy to mention that we attempted to attain 2D NMR of samples but their spectra did not produce accurate results. Overall, artificial selenization changed the configuration of monosaccharide residues in MPS.

### 2.3. Property Analysis

**Figure 5:**
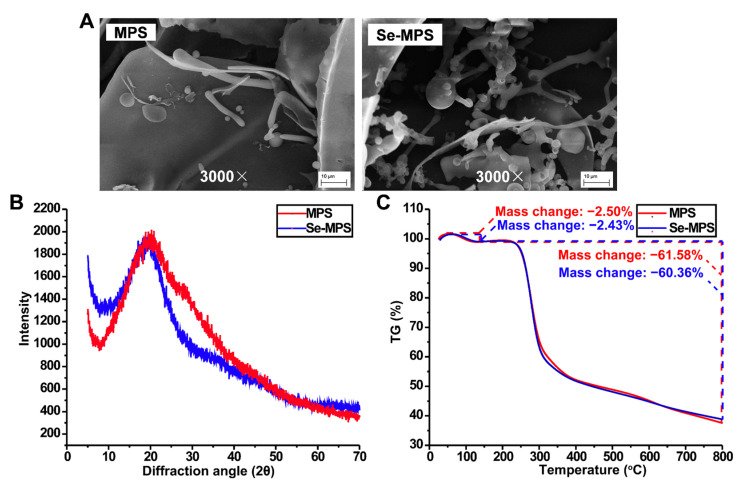
SEM (**A**), XRD (**B**), and TG (**C**) analysis of MPS and Se-MPS.

The SEM images of MPS and Se-MPS (Figure 5A) showed that the two samples exhibited noticeable differences in the apparent morphology. More specifically, MPS was mainly in smooth and dense flakes with little irregular filaments or rods, while Se-MPS was largely irregular with filaments, dendrites, and small fragments. Generally, filamentous and fragmented morphology can increase the specific surface area of polysaccharides and enhance their bioactivity [34]. Our results indicated that selenization increased the inhomogeneous surface distribution of MPS and further reduced the interactions and cross-links between polysaccharides, which might be due to surface changes caused by intermolecular or van der Waals interactions under the Se interaction [8,35].

XRD analysis (Figure 5B) showed that MPS and Se-MPS had a single diffraction peak at 21^o^ with broad and deep intensity. However, the diffraction peak of MPS was broader and more intense than Se-MPS, which indicated its easier crystallization [8]. This result was also consistent with SEM analysis.

TG analysis (Figure 5C) showed that both MPS and Se-MPS had a weight loss in the temperature range of 25–200 °C and 200–800 °C. Less weight loss in the temperature range of 25–200 °C could be attributed to the loss of physical adsorption of polysaccharides to water, and more weight loss at 200–800 °C could be due to depolymerization of polysaccharides and cleavage of C-O/C-C in sugar ring units. The total weight loss of MPS and Se-MPS was 64.08% and 62.79%, respectively, indicating that the structural stability of Se-MPS might be higher than that of MPS. It has been reported that other substances bound to polysaccharides could affect their thermal stability [36], and the binding of Se to MPS accordingly caused changes in the thermal stability.

### 2.4. Immunomodulation Analysis

**Figure 6:**
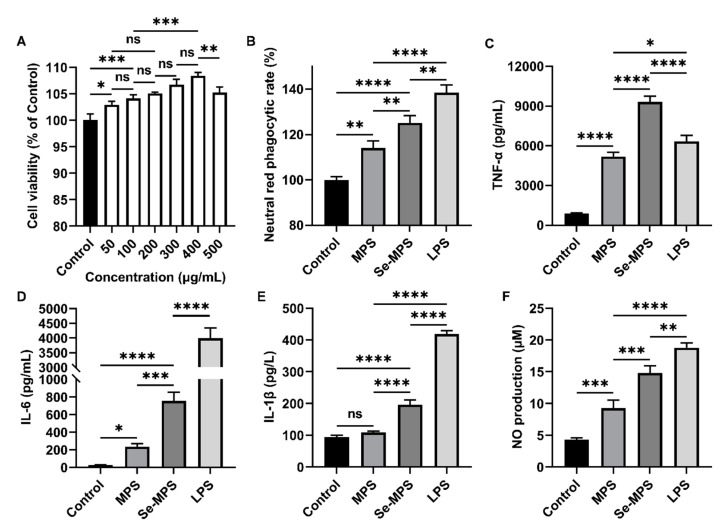
Cell viability of RAW 264.7 cells treated with Se-MPS (**A**), neutral red phagocytic rate (**B**), TNF-α (**C**), IL-6 (**D**), IL-1β (**E**), and NO levels (**F**) of RAW 264.7 cells treated with MPS and Se-MPS. *, *p* < 0.05; **, *p* < 0.01, ***, *p* < 0.001; ****, *p* < 0.0001; ns, not significant; MPS—*Morel* polysaccharide; Se-MPS—Se-enriched *Morel* polysaccharide; LPS—lipopolysaccharide.

Edible fungi contain various compounds with immunomodulatory properties, and polysaccharides are among their key active ingredients. These compounds participate in regulating various aspects of the immune system, including immune organs, cells, cytokines, and receptors, and they also have a positive effect on human immunity [37]. Innate immune regulation is critical to the host’s ability to respond rapidly against pathogens. As an essential member of the host immune defense system, macrophages play a crucial role in the innate immune response, serving as key directors of host inflammation and other immune processes. They can co-operate with different types of cells to resist external adverse factors as an essential part of innate cellular immunity [38]. Activation of macrophages is considered a major and essential step in immune system stimulation [39]. As shown in Figure 6A, Se-MPS treatment did not show cytotoxicity and could enhance the viability of RAW 264.7 cells in a dose-dependent manner, with the optimum effect at 400 μg/mL. Additionally, we further explored the regulation of the phagocytosis of RAW 264.7 cells by the two polysaccharide samples. Our results showed that co-incubation with Se-MPS or MPS significantly promoted the phagocytosis of RAW 264.7 cells (Figure 6B). Phagocytosis is an important barrier for host cells to exert their innate immune system, and it is one of the most important features of macrophage activity [40]. Macrophages improve the body’s anti-infection ability by phagocytizing invading pathogens and aging and aberrant cells [41]. In addition to regulating cell viability and phagocytosis, the treatment of Se-MPS and MPS significantly promoted the release of critical immunomodulatory cytokines (TNF-α, IL-6, and IL-1β) and greatly facilitated the release of NO secreted (Figure 6C–F). TNF-α, IL-1β, and IL-6 are cytokines essential for regulating immune responses, an antigen presentation function, phagocytosis, and innate immunomodulation of macrophages [42]. The positive immunomodulation of macrophages is mainly achieved by their phagocytosis and secretion of some cytokines (IL-1, IL-6, and TNF-α), and the generation of NO can enhance their phagocytosis [43]. TNF-α is a crucial cytokine of inflammation in host defense, and it can promote the expression of immune regulatory factors in the body and then enhance the phagocytosis of pathogenic micro-organisms by macrophages to exert immunomodulatory activity [44]. In addition, it can promote the expression of IL-1β by macrophages, thereby enhancing cell activity and amplifying the inflammatory response. IL-1β/6 plays a vital role in the transition from innate to adaptive immune responses and can be released by activated macrophages [45]. As an essential regulator of host defense responses, IL-1β/6 is involved in metabolism in various inflammatory and immune diseases [45,46]. In addition, IL-1β can also promote the secretion of IL-6 and TNF-α [47]. The higher the concentration of TNF-α and IL-1β/6, the stronger the function of macrophages and the body’s disease resistance. NO is a non-specific effector gas molecule synthesized by iNOS, which plays a vital role in regulating the apoptosis of tumor cells and inhibiting the growth of various pathogenic micro-organisms [48]. When macrophages are stimulated by pathogens and micro-organisms, they will express iNOS and produce a large amount of NO [49]. Therefore, NO is the fundamental prerequisite for macrophages to perform phagocytosis, and its secretion has become one of the evaluation indicators of macrophage immune activity. Our results revealed that Se-MPS and MPS could remarkably activate RAW264.7 macrophages to enhance the release of pro-inflammatory cytokines (TNF-α and IL-1β/6) and the secretion of NO to enhance macrophage cell activity and phagocytosis, which was consistent with previously reported immunomodulatory effects of other edible fungal polysaccharides [37].

**Figure 7:**
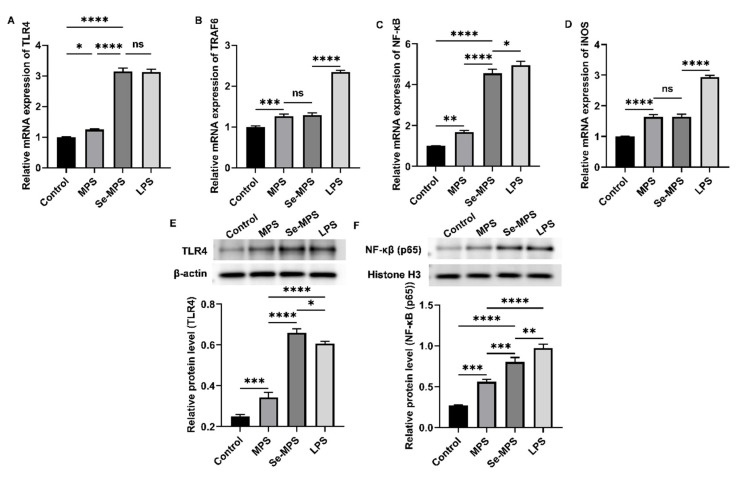
Key mRNA expression levels of TLR4 (**A**), TRAF6 (**B**), NF-κB (**C**), and iNOS (**D**), and protein expression levels of TLR4 (**E**) and NF-κB (**F**) in RAW 264.7 cells treated with MPS and Se-MPS. *, *p* < 0.05; **, *p* < 0.01, ***, *p* < 0.001; ****, *p* < 0.0001; ns, not significant; MPS—*Morel* polysaccharide; Se-MPS—Se-enriched *Morel* polysaccharide; LPS—lipopolysaccharide.

**Figure 8:**
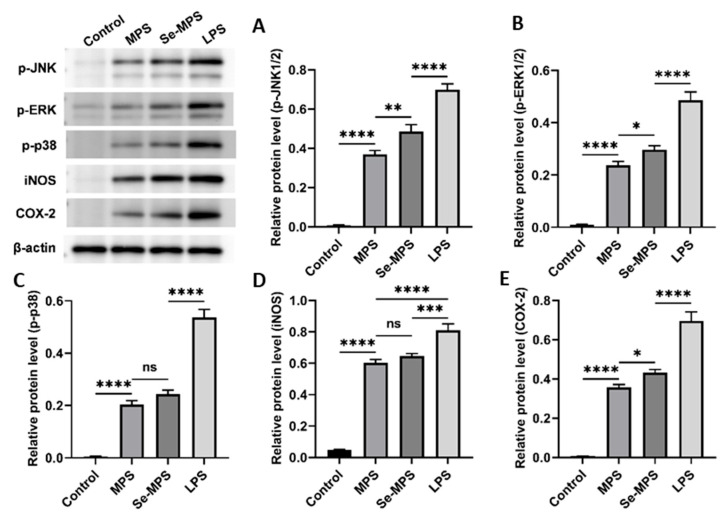
p-JNK (**A**), p-ERK (**B**), p-p38 (**C**), iNOS (**D**), and COX-2 (**E**) protein expression levels in RAW 264.7 cells treated with MPS and Se-MPS. *, *p* < 0.05; **, *p* < 0.01; ***, *p* < 0.001; ****, *p* < 0.0001; ns, not significant; MPS—*Morel* polysaccharide; Se-MPS - Se-enriched *Morel* polysaccharide; LPS—lipopolysaccharide; p-JNK—phosphorylated c-Jun N-terminal kinase; p-ERK—phosphorylated extracellular signal-regulated kinase; p-p38—phosphorylated protein-38; iNOS—inducible nitric oxide synthase; COX-2—cyclooxygenase-2.

Previous studies have demonstrated that polysaccharides can recognize specific pattern recognition receptors (such as TLR4) on the macrophages, modulate the expression of these receptors, activate immune signaling pathways, and enhance macrophage phagocytosis and the release of cytokines such as IL-6, TNF-α, and IL-1β [50]. Therefore, our study aimed to examine the mechanisms by which Se-MPS and MPS improve macrophage activity and phagocytosis and regulate related cytokines. Our results showed that both Se-MPS and MPS treatment significantly increased the mRNA expressions of TLR4, TRAF6, NF-κB, and iNOS (Figure 7A–D), and the protein expression of TLR4, NF-κB (p65), p-JNK, p-ERK, p-p38, iNOS, and COX-2 (Figure 7E,F and Figure 8). TLR4, a crucial membrane receptor expressed on the surface of macrophages, mediates the activation of macrophages by transforming extracellular signals [51]. An experiment shows that a polysaccharide G1-4A could activate macrophages through the TLR4 pathway, and macrophage activity may be reduced after blocking TLR4 action using siRNA and antibodies [52]. After TLR4 is activated, it binds to a ligand to form a complex between the cytoplasmic region and myeloid differentiation primary response gene 88 (Myd88), activates tumor necrosis factor receptor-associated molecule 6 (TRAF6), and finally initiates the expression of mitogen-activated protein kinase (MAPK) signaling pathway [53]. The MAPK pathway has been reported to play a key role in TLR4 signaling and the production of pro-inflammatory mediators [43]. The MAPK signaling pathway, mainly composed of JNK1/2, ERK1/2, and p38, mediates the activation of macrophages and the expression of inflammatory-related genes [54]. The activated MAPK signaling pathway further mediates and activates the NF-κB pathway to regulate immune response [55]. The NF-κB protein family can selectively bind to IκB to regulate the expression of many genes and participate in the response of cells to external stimulation [56]. The promoters of the immunomodulatory cytokine genes, including TNF-α, IL-1β, and IL-6, all have corresponding binding sites for NF-κB, and thus they are regulated by NF-κB activity [57]. In addition to pro-inflammatory cytokines, studies have shown that NO production is regulated by NF-κB [58]. iNOS and COX-2 are critical in the regulation of NO and are key mediators in many inflammatory conditions [59]. Our results show that Se-MPS and MPS could produce pro-inflammatory cytokines via activating the TLR4-TRAF6-MAPKs-NF-κB cascade signaling pathway, which was similar to the other report on the immunomodulatory activity of MPS [60]. Furthermore, we found that Se-MPS had a better regulatory effect than MPS, suggesting that selenized MPS could also exert immunomodulatory activity via this mechanism, similar to the previously reported mechanism through which selenized polysaccharides exert immunomodulation [61]. As for the better regulation effect of Se-MPS, we speculate that Se might be absorbed and utilized by macrophage cells to participate in and enhance the transduction of immunomodulatory signals in cells [62]. In addition, the unique chemical composition and structural properties of Se-MPS contributed to its stronger immunomodulatory activity.

## 3. Materials and Methods

### 3.1. Chemicals and Reagents

The *Morchella esculenta* (L.) Pers. strain (ACCC50764) was provided from the Agricultural Culture Collection of China (ACCC) (Beijing, China). The murine macrophage-like cell line RAW 264.7 was gifted from Shanghai Normal University (Shanghai, China). Diethylaminoethyl (DEAE) Sepharose Fast Flow gel was obtained from GE Co. (St. Louis, MO, USA). The fetal bovine serum (FBS) and Dulbecco’s modified Eagle’s medium (DMEM) culture medium were provided by Hyclone Co. (Logan, UT, USA). The endotoxin lipopolysaccharide (LPS) was provided by Sigma Co. (St Louis, MO, USA). Tumor necrosis factor (TNF-α), interleukin 6 (IL-6), interleukin-1β (IL-1β), and CCK8 detection kits were purchased from Nanjing Jiancheng Bioengineering Institute (Nanjing, China). Neutral red and Griess reagents were provided by MACKLIN Co. (Shanghai, China). Penicillin–Streptomycin was obtained from Thermo Fisher Scientific (Waltham, MA, USA). Toll-like receptor 4 (TLR4), phosphorylated protein-38 (p-p38), and phosphorylated c-Jun N-terminal kinase (p-JNK1/2) polyclonal antibodies were obtained from Santa Cruz Co. (Santa Cruz, CA, USA). Nuclear factor kappa-B p65 (NF-κB p65) and phosphorylated extracellular signal-regulated kinase (p-ERK1/2) were provided by CST Co. (Danvers, MA, USA). Inducible nitric oxide synthase (iNOS) and cyclooxygenase-2 (COX-2) monoclonal antibodies were obtained from Abcam Co. (Boston, MA, USA). All other chemicals and solvents were of analytical grade.

### 3.2. Polysaccharides Preparation and Purification

A total of 15 mL of *Mercella* liquid strand was inoculated into 150 mL potato dextrose agar (PDA) liquid medium (containing 20 μg/mL sodium selenite) or ordinary PDA liquid medium with a 170 r/min shake cultivation at 26 °C for five days. After that, the selenium-enriched or ordinary mycelium was filtered, collected, and freeze-dried after liquid fermentation. Next, 1500 mL of boiling water was added to the ground mycelium (50 g) and extracted for 2 h. The precipitation after centrifugation at 4000 r/min for 10 min was repeatedly extracted twice. Firstly, the collected supernatant was combined and concentrated with a rotary evaporator, then three volumes of 95% ethanol were added and stood overnight at 4 °C. After 10 min of centrifugation (4000× *g*), the precipitation was freeze-dried to obtain crude Se-MPS or MPS. A total of 2 g of crude polysaccharide was dissolved in 0.02 M phosphate-buffered saline (PBS) over 0.45 μm filter membrane, and then it was eluted by DEAE Sepharose Fast Flow ion exchange column (2.6 cm × 50 cm). The eluent was 0~2 M NaCl solution (0, 0.5, 1, 1.5, and 2 M NaCl) with a flow rate of 1 mL/min. The anthrone–sulfuric acid method was employed to track the elution process. The eluent was collected and dialyzed by a dialysis bag (cut-off 10 kDa) for 36 h. The purified polysaccharide sample was obtained by freeze-drying.

### 3.3. Microchemical Characterization

#### 3.3.1. Preliminary Composition Analysis

The total sugar content was analyzed by the anthrone–sulfuric acid method [63]. The uronic acid content was measured by the meta-hydroxyphenyl method [64]. The protein content was determined by the Bradford method [65]. The selenium content was estimated by atomic fluorescence spectrometry [66].

#### 3.3.2. Monosaccharide Composition

A modified monosaccharide composition for the purified polysaccharide was carried out based on the report published by Zhu et al. [8,67]. Briefly, approximately 5 mg of sample was hydrolyzed with 2 M trifluoroacetic acid for 2 h at 121 °C in a sealed tube. Next, the sample was dried with nitrogen, and methanol was repeatedly added to wash the sample. For measurement, the dried residue was redissolved in deionized water and filtered through a microporous filtering film with a mesh size of 0.22 μm. The sample was analyzed by high-performance anion-exchange chromatography (HPAEC, Thermo Fisher Scientific, Waltham, MA, USA) on a CarboPac PA-20 anion-exchange column (150 mm × 3.0 mm, Dionex) using a pulsed amperometric detector (PAD, Dionex ICS 5000 system) by Sanshu Biotech. Co., LTD (Shanghai, China). A similar procedure was followed for measuring monosaccharide standards, including fucose (Fuc), rhamnose (Rha), arabinose (Ara), galactose (Gal), glucose (Glc), xylose (Xyl), mannose (Man), fructose (Fru), ribose (Rib), galacturonic acid (GalA), glucuronic acid (GulA), mannuronic acid (GlcA), and guluronic acid (ManA).

#### 3.3.3. Molecular Weight (Mw) Distribution

A procedure developed by Zhu et al. [6] was used to assess homogeneity and Mw distribution. In short, high-performance gel permeation chromatography (HPGPC) (Waters1515, Waters, Milford, MA, USA) equipped with a differential refractive index detector (Waters2410, Waters, Milford, MA, USA) and the series column (8 × 300 mm) (polymer matrix water-soluble SEC (GFC) column OHpak SB-803 HQ, Ohpak SB-804 HQ, and Ohpak SB-805 HQ) was employed to evaluate the weight-average (Mw). The sample concentration was 5 mg/mL, the flow rate was 0.6 mL/min, the injection volume was 30 μL, and the column temperature was kept at 40 °C. A standard curve was established by calibrating the column with dextran standards (10–1000 kDa).

#### 3.3.4. Ultraviolet-Visible (UV-vis) and Fourier-Transform Infrared (FT-IR) Analysis

A UV-vis spectrophotometer (Pussy General Instruments, Beijing, China) was used to scan the polysaccharide sample (80 g/mL) in the range of 200–700 nm.

An infrared spectrometer (PE-1730, Massachusetts, USA) was used to systematically scan the PBS sample with 100 mg of KBr powder over a wavenumber range of 4000–500 cm^−1^.

#### 3.3.5. Raman Spectroscopy Analysis

Raman spectra were recorded by using a HORIBA Labram HR800 Raman spectrometer (Kyoto, Japan) with a scanning range of 1700~300 cm^−1^. The excitation source was a He–Cd ion laser with an excitation wavelength of 785 nm, a laser power of 100 mW, 5–10 scans with a spectral resolution of 5 cm^−1^, and an exposure time of 10–20 s.

#### 3.3.6. Nuclear Magnetic Resonance (NMR) Spectroscopy Analysis

A total of 20 mg of polysaccharide sample was dissolved in D_2_O, repeatedly lyophilized three times, and dissolved in 0.5 mL of D_2_O. ^1^H and ^13^C NMR spectra were obtained by Avance III HD spectrometer (Bruker, Billerica, MA, USA).

#### 3.3.7. Scanning Electron Microscopy (SEM) Analysis

The polysaccharide sample (2 mg) was attached to a metal block with conductive adhesive, and gold was sprayed on its surface. Then its apparent morphology (3000×) was analyzed by a HITACHSU8010 scanning electron microscope (JEOL, Tokyo, Japan).

#### 3.3.8. X-ray Diffraction (XRD) Analysis

X-ray diffractometer D8 ADVANCE (Bruker, Billerica, MA, USA) was used to measure the sample. As for the X-ray diffraction conditions, 35 kV of tube pressure, 100 mA of tube current, 5~70° angle, and 0.05° angle gradient were applied.

#### 3.3.9. Thermogravimetric (TG) Analysis

A simultaneous thermal analyzer (NETZSCH STA 449F3, Selb, Free State of Bavaria, Germany) was used to determine the thermal stability of polysaccharide samples. Under nitrogen protection, the sample (10 mg) was heated at 10 °C/min and observed between 30 and 800 °C.

### 3.4. Immunomodulatory Evaluation

#### 3.4.1. Cell Viability

RAW 264.7 cells in the exponential growth phase were seeded in a 96-well plate at a density of 5 × 10^5^ cells/well and cultured with DMEM medium containing 10% FBS and penicillin–streptomycin (100 U/mL) for 2 h. Next, a series of concentration gradients of Se-MPS samples (50, 100, 200, 300, 400, and 500 μg/mL) was added and incubated for 24 h, and the untreated group was regarded as the Control group. After that, cell viability was then determined using a CCK8 kit.

#### 3.4.2. Evaluation of Cellular Phagocytosis

RAW 264.7 cells were seeded in a 96-well plate at a density of 5 × 10^5^ cells/mL and incubated with samples (400 μg/mL MPS, 400 μg/mL Se-MPS, and 1 μg/mL LPS, respectively) in a humidified environment (37 °C, 5% CO_2_, and 95% humidity). The Control group was replaced with the corresponding PBS. After incubation for 24 h, the medium was removed and 100 μL of 1% neutral red solution was added to each well. After incubation for 30 min, cells were washed with PBS three times, and 200 μL of cell lysate (equal volume of absolute ethanol and acetic acid mixture) was added. After standing overnight at 4 °C, the absorbance was measured at 540 nm. The phagocytic rate of cells was calculated using the following formula:

Neutral red phagocytic rate (%) = (A_Sample_/A_Control_) × 100% (1)

#### 3.4.3. Biochemical Parameters

The levels of TNF-α, IL-6, and IL-1β and NO content in the cell-free supernatant were measured by using commercially available kits.

#### 3.4.4. Real-Time PCR Assay

The total RNA was extracted from cells and reverse transcribed into cDNA using Servicebio^®^RT First Strand cDNA Synthesis Kit instructions (Service, Wuhan, China). Following the light quantitative PCR kit instructions, mRNA expression was assessed using SYBR qPCR Master Mix (High ROX, Wuhan, China), which contained the following primers:

Primer TLR4 Forward: CTGGGTGAGAAAGCTGGTAA
Primer TLR4 Reverse: AGCCTTCCTGGATGATGTTGG
Primer TRAF6 Forward: CATCTTCAGTTACCGACAGCTCAG
Primer TRAF6 Reverse: TGGTCGAGAATTGTAAGGCGTAT
Primer NF-κB Forward: CCAAAGAAGGACACGACAGAATC
Primer NF-κB Reverse: GGCAGGCTATTGCTCATCACA
Primer iNOS Forward: CAACCAGTATTATGGCTCCT
Primer iNOS Reverse: GTGACAGCCCGGTCTTTCCA
Primer β-actin Forward: TGGAATCCTGTGGCATCCATGAAAC
Primer β-actin Reverse: TAAAACGCAGCTCAGTAACAGTCCG

#### 3.4.5. Western Blot Assay

After separating total proteins from cells, they were electroblotted onto PVDF membranes. Following 1 h of blocking in 5% BSA, the primary antibodies (1:500–1:1000 dilution) were incubated overnight at 4 °C. An image analyzer quantitative system (Stepone plus, ABI, Waltham, MA, USA) was used to visualize the conjugates after 30 min at room temperature incubation with a secondary antibody labeled with horseradish peroxidase. AlphaEase FC software (Alpha Innotech, San Leonardo, CA, USA) was used to perform a quantitative grayscale analysis of protein expression.

### 3.5. Statistical Analysis

The data are presented as means with standard deviations in at least three replicates. In addition, a one-way ANOVA with the Tukey test for multiple groups was carried out using GraphPad Prism 8.0 (GraphPad Software Inc., San Diego, CA, USA). *p*-values less than 0.05 were considered significant.

## 4. Conclusions

In this study, selenization modifications altered the microchemical composition and structural properties of *Morel* polysaccharides. In addition, the selenium-enriched *Morel* polysaccharide activated the TLR4-TRAF6-MAPKs-NF-κB cascade signaling pathway, thereby enhancing the activity and phagocytosis of macrophages and secreting pro-inflammatory cytokines to exert immunomodulatory functions. Our results reveal that selenium-enriched *Morel* polysaccharide is a potential natural immune system enhancer and could be used as food or medicine to enhance the body’s immunity. In addition, our results contribute to a better understanding of the structural properties and functional activities of selenium-enriched *Morel* polysaccharides. However, characterization of its fine structure was not represented in this work, and further exploration of its structure and more targets related to its immunomodulation is needed in the future.

## Figures and Tables

**Table 1:** Preliminary chemical composition of *Morel* polysaccharide (MPS) and Se-enriched *Morel* polysaccharide (Se-MPS).

Sample	Total Sugar (%)	Uronic Acid (%)	Protein (%)	Se (μg/g)	Yield (%)
MPS	87.8 ± 0.32	0.73 ± 0.03	0.92 ± 0.06	2.71 ± 0.05	11.68 ± 0.25
Se-MPS	98.6 ± 0.22	0.37 ± 0.01	0.32 ± 0.02	214.07 ± 0.78	14.65 ± 0.18

## Data Availability

The datasets used and/or analyzed during the current work are available from the corresponding author on request.

## Supplementary Materials

The following supporting information can be downloaded at: https://www.mdpi.com/article/10.3390/molecules28072885/s1, Figure S1: Elution curves of crude MPS and Se-MPS.
