# Supplementary material for: Microanalysis Characterization and Immunomodulatory Effect for Selenium-Enriched Polysaccharide from Morchella esculenta (L.) Pers
Source: Molecules. 2023 Mar 23;28(7):2885. doi: 10.3390/molecules28072885 (PMC10096435; doi:10.3390/molecules28072885)
Supplement: Supplementary file 1 [file molecules-28-02885-s001.zip › molecules-2233096-supplementary.pdf]

Supplementary Materials

Microanalysis Characterization and Immunomodulatory Effect for Selenium-Enriched Polysaccharide from *Morchella esculenta* (L.) Pers.

Lijuan Qian [1,†], Mengxiang Du [1,†], Xiaoyan Yang [1], Qian Wang [2], Shengwei Huang [2], Yuhan Ma [2] and Yujun Sun [2,*]

1 College of Agriculture, Anhui Science and Technology University, Fengyang 233100, China

2 College of Life and Health Sciences, Anhui Science and Technology University, Fengyang 233100, China

* Correspondence: sunyujun208@126.com

† These authors contributed equally to this work.

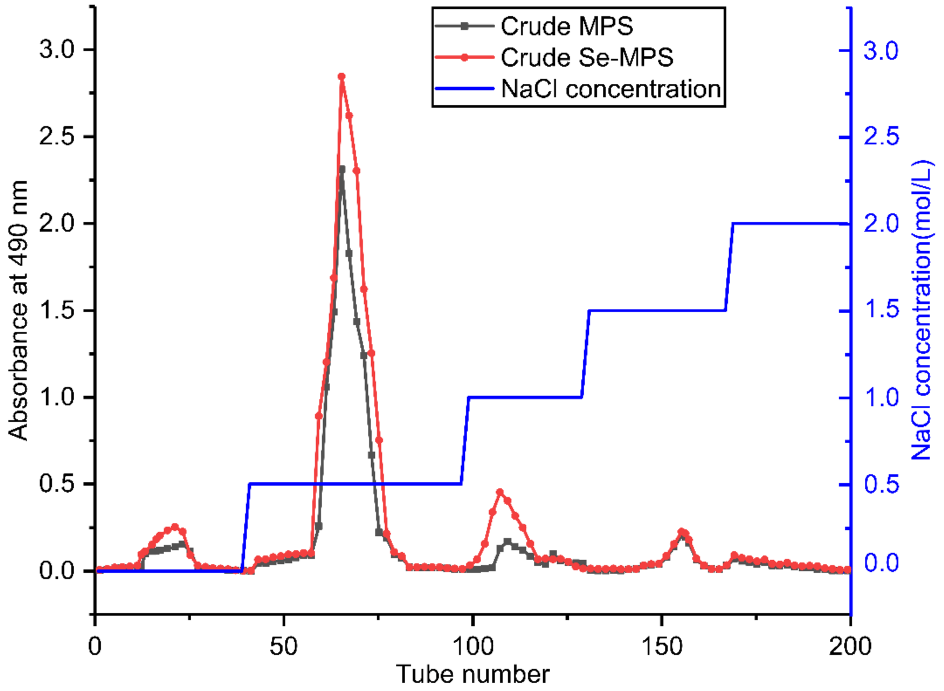

Figure S1. Elution curves of crude MPS and Se-MPS.
